# Renal transplant anastomotic time–Every minute counts!

**DOI:** 10.3389/fmed.2022.1024137

**Published:** 2023-01-18

**Authors:** Nikhil Mahajan, Munish K. Heer, Paul R. Trevillian

**Affiliations:** ^1^Newcastle Transplant Unit, Division of Surgery, John Hunter Hospital, New Lambton Heights, NSW, Australia; ^2^Hunter Transplant Research Foundation, Hunter Medical Research Institute, New Lambton Heights, NSW, Australia

**Keywords:** renal transplant, anastomotic time, delayed graft function, donation after brain and cardiac death, cold ischemia time

## Abstract

The impact of anastomotic time in renal transplant is under recognized and not well studied. It is one of the few controllable factors that affect the incidence of delayed graft function (DGF). Our study aimed at quantifying the impact of anastomotic time. We performed a retrospective review of 424 renal transplants between the years 2006 and 2020. A total of 247 deceased donor renal transplants formed the study cohort. Patients were divided into two groups based on the presence or absence of DGF. Variables with *p* < 0.3 were analyzed using the binary logistic regression test. The final analysis showed anastomotic time to be significantly associated with DGF with odds ratio of 1.04 per minute corresponding to 4% increase in DGF incidence with every minute increment in anastomotic time. Other variables that had significant impact on DGF were DCD donor (odds ratio – 8.7) and donor terminal creatinine. We concluded that anastomotic time had significant impact on the development of DGF and hence should be minimized.

## 1. Introduction

Renal transplant outcomes have come of age since its inception. With the improved immunosuppression and better understanding of the factors that affect the transplanted graft, the 5-year graft survival in deceased donor renal transplant has improved from 35–40% in the mid-1980s to 85–90% in the last decade (1–3). Despite this improvement, delayed graft function (DGF) has proven to be the Achilles heel and remains the most encountered complication in the immediate post-transplant period (4, 5). The incidence of DGF has been on the rise with the acceptance of marginal donors and ranges from 20% to as high as 70% in deceased donor transplant (6–9).

The deleterious effects of DGF on graft survival are well established since the first reference paper published by Anderson et al. in 1979 showing inferior long-term graft survival in patients with DGF (10) still holds true, and similar results continue to be validated in other studies (11, 12).

The cause of DGF is multifactorial and includes many non-modifiable donor and recipient factors (13). Ischemia times are perhaps the only controllable factors that affect DGF incidence. There are three critical ischemia times involved in renal transplant namely donor warm ischemia (DWIT), cold ischemia time (CIT), and the recipient warm ischemia time or anastomotic time (AT). AT and its impact on graft function have been sparingly studied in comparison to DWIT and CIT (14–16). AT is the time period between the removal of the kidney from ice to reperfusion.

The objective of our study was to evaluate the impact of AT on the incidence of DGF.

## 2. Materials and methods

### 2.1. Study design

Our study design was a retrospective chart review of consecutive renal transplant recipients at our center from 2006 to 2020. Data were extracted from prospectively recorded electronic health records of our departmental “Transnet” and other data repositories in our hospital. Ethics approval was obtained from our hospital ethics committee.

### 2.2. Patient demographic and patient selection

Recipient and donor data were extracted that included demographic, comorbidities, donor type, kidney donor profile index (KDPI), CIT, AT, human leucocyte antigen mismatch (HLA MM), post-transplant dialysis incidence, and glomerular filtration rate (GFR) at 1 month and 1 year (mean GFR between 180 and 365 days).

**Inclusion criteria**—All deceased donor renal allograft transplants performed at John hunter hospital (JHH) between 2006 and 2020 were included in the study.

**Exclusion criteria**—Recipients of dual kidney transplants including en bloc kidneys and patients who suffered early graft loss within the first 7 days post-transplant were excluded.

### 2.3. Immunosuppression protocol

Standard induction immunosuppression included basiliximab 20 mg on day 0 and day 4 and methylprednisolone 500 mg at induction of anesthesia. Anti-thymocyte globulin (ATG) was used only in high immunological-risk patients. Standard maintenance immunosuppression included tacrolimus, mycophenolate mofetil, and prednisolone. All patients received pneumocystis and cytomegalovirus (CMV) prophylaxis.

### 2.4. Statistical analysis

Delayed graft function was defined as the requirement of hemodialysis in the first-week post-transplant. Patients were divided into two groups based on the presence or absence of DGF. All analyses were done using SAS academic edition. Continuous variables were presented as the median and interquartile range (IQR) as they were not normally distributed. Categorical variables were expressed as numbers and percentages. Non-parametric tests, such as Mann–Whitney *U*-test, were used to compare continuous variables, and the Pearson chi-square test was used for categorical variables between groups. Binary log regression was used to analyze the impact of AT on DGF. Other variables used in the model were chosen based on prior knowledge and a *p*-value of < 0.3 on univariate tests to account for potential confounders. The other covariates included in the model were the age and sex of the recipient, donor age, CIT, and donation after cardiac death (DCD) donor status. There were only 14 missing values, which were imputed by the “maximum likelihood estimation method.” Model fitting was tested using the Hosmer and Lemeshow goodness-of-fit test. The c-statistic of the final model was 0.8.

## 3. Results

### 3.1. Patients

**FIGURE 1:**
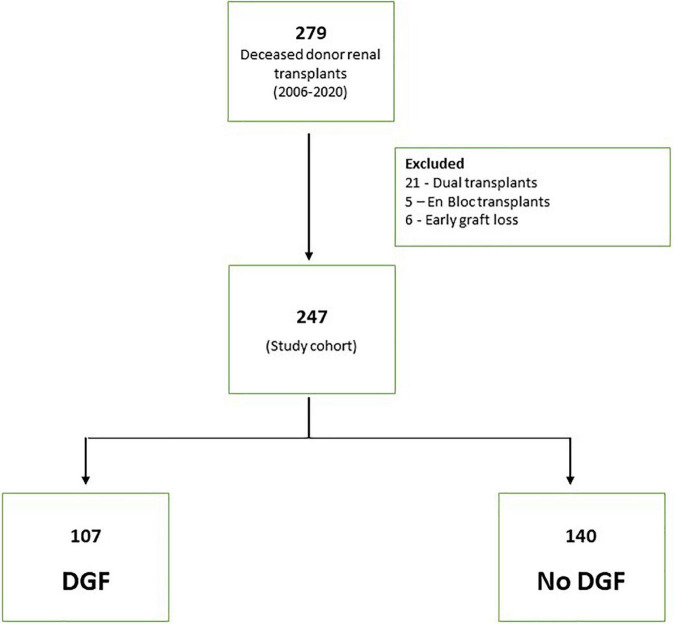
Consort diagram of study cohort.

A total of 279 deceased donor renal transplants were done between 2006 and 2020. The final study cohort consisted of 247 transplant after excluding 32 patients (21 with dual transplants, 5 enbloc transplants and 6 early graft losses) (Figure 1). The characteristics of the patients are shown in Table 1. A total of 62.3% of recipients were men. The median age of the recipient was 54 years (IQR 43, 63). The median donor age was 52 years (IQR 37, 62). In total, 26.7% (66/247) of donors were DCD. Notably, 21 (8.5%) donors were diabetic and 67 (27.1%) were hypertensive. The median WIT in DCD donors was 19 min (IQR 14, 32). The median AT was 40 min (IQR 33, 48). In total, 16 out of 247 (6.5%) patients had two warm arterial anastomoses. Anastomotic time (median 45 IQR 39.5, 47.5) was not statistically different in these patients compared to patients with one warm arterial anastomosis (median 40, IQR 33, 48) *p* = 0.108. Median CIT was 840 min (IQR 660, 980). Around 46% of recipients were well matched with the donors having 0–2/6 HLA MM.

**TABLE 1:** Summary of the recipient, donor, and transplant variables in the study cohort (*n* = 247).

	Value
**Recipient variables**	
Recipient age (median, IQR)	54 (43–63)
Recipient sex–Male (*n*%)	154/247 (62.3%)
Dialysis vintage–Months (median, IQR)	48 (27–81)
**Donor variables**	
Donor age (median, IQR)	52 (37–62)
Donor sex–Male (*n*%)	115/247 (46.6%)
DCD donor (*n*%)	66/247 (26.7%)
Donor terminal creatinine (median, IQR) μmol/L	71.5 (56.5–105)
Donor comorbidity (*n*%)	
Diabetic	21/247 (8.5%)
Hypertension	67/247 (27.1%)
KDPI (median, IQR)	44 (19.5–69.5)
**Transplant variables**	
HLA mismatch on A, B and DR (*n*%)	
0/6	19 (7.7%)
1/6	24 (9.7%)
2/6	73 (29.6%)
3/6	21 (8.5%)
4/6	38 (15.4%)
5/6	43 (17.4%)
6/6	29 (11.7%)
Cold ischemia time (minute) (median, IQR)	840 (660–980)
Anastomosis time (minute) (median, IQR)	40 (33–48)
GFR at 1 month (median, IQR) ml/min	37 (25–45.5)
GFR 1 year (median, IQR) ml/min	40 (30.5–51.5)

### 3.2. Delayed graft function

**FIGURE 2:**
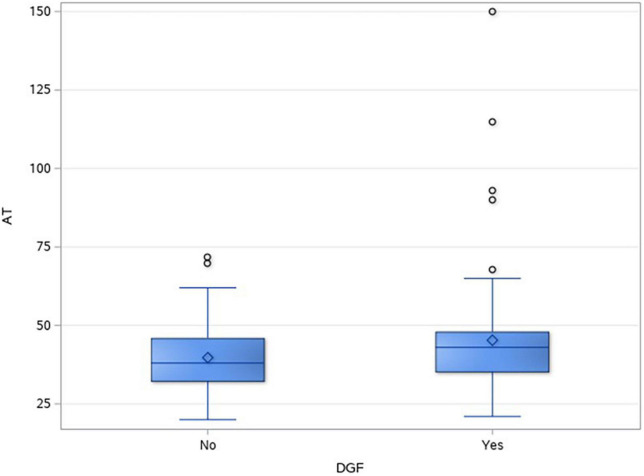
Box and whisker plot showing the distribution of anastomotic time in the two groups.

In total, 43.3% (107/247) of DD renal transplants developed DGF. Table 2 shows the different factors and their impact on DGF. In the univariate analysis, DCD recipients (49/66, 74.2%) had a significantly higher incidence of DGF as compared to donation after brain death (DBD) recipients (58/181, 32%), *p* = < 0.001. Anastomotic time was found to be significantly longer in the DGF group, *p* = 0.008. Figure 2 shows the box and whisker plot of AT and its impact on DGF.

**TABLE 2:** Summary of the recipient, donor, and transplant variables and their impact on DGF.

	DGF (*N* = 107)	No DGF (*N* = 140)	*P*-value
**Recipient variables**			
Recipient age (median, IQR)	54 (43–61)	54 (42.5–63)	0.618
Recipient sex (male%)	74 (69.2%)	80 (57.1%)	0.053
Dialysis vintage in months (median, IQR)	54 (32–85)	45 (24–77)	0.03
**Donor variables**			
Donor age (median, IQR)	54 (40–64)	50.5 (36–62)	0.209
Donor sex (male%)	50 (46.7%)	65 (46.4%)	0.359
DCD donor (*n%*)	49 (45.8%)	17 (12.1%)	<0.001
Terminal donor creatinine (μmol/L) (median, IQR)	77.5 (56–128.5)	69 (57–91.5)	0.107
Donor comorbidity (*n*%)			
Diabetes	8	13	0.613
Hypertension	30	37	0.778
KDPI (median, IQR)	42 (18–70)	47 (23–70)	0.555
**Transplant variables**			
HLA mismatch on A, B, and DR (*n*%)			
0/6	8	11	
1/6	7	17	
2/6	24	49	
3/6	13	8	0.078
4/6	18	20	
5/6	20	23	
6/6	17	12	
CIT (minute) (median, IQR)	840 (665–1,020)	826.5 (620.5–952.5)	0.250
AT (minute) (median, IQR)	43 (35–48)	38 (32–56)	0.008

### 3.3. Binary logistic regression analysis

Binary logistic regression analysis showed AT to be significantly associated with DGF with an odds ratio of 1.04 per minute (*p* = 0.002). DCD transplants had an 8.7% higher chance of developing DGF as compared to DBD transplants. Other confounders that had a significant effect on the outcome were donor age and terminal donor creatinine. CIT and recipient sex were not found to have significant differences (Table 3).

**TABLE 3:** Determinants of DGF by binary logistic regression analysis.

Independent variable	Regression coefficient	Standard error	Wald chi-square	*P*-value	Odds ratio (95% CI)
AT	0.040	0.013	9.978	0.002	1.042 (1.015–1.067)
DCD	2.1705	0.350	38.315	<0.001	8.762 (4.40–17.4)
Donor age	0.020	0.009	4.72	0.03	1.021 (1.002–1.040)
Terminal donor creatinine	0.00687	0.00169	16.458	<0.001	1.007 (1.004–1.010)

### 3.4. DGF and GFR

**FIGURE 3:**
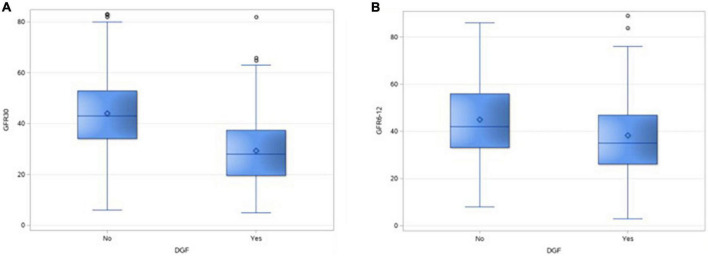
Box and whisker plot showing the distribution of GFR of 30 days **(A)** and at 1 year **(B)** in the two groups.

There was a significant difference in the GFR at 1 month and 1 year in DGF and non-DGF groups (Table 4), suggesting the adverse impact of AT on short- and median-term graft function (Figures 3A, B). There were five missing values of GFR at 1 month and 34 missing values for creatinine at 1 year.

**TABLE 4:** Association of DGF and graft function.

Graft function	DGF	No DGF	*P*-value
GFR at 1 month (median, IQR) ml/min	28 (19.5–37.5)	43 (34–53)	<0.001
GFR at 1 year (median, IQR) ml/min	35 (26–47)	42 (33–56)	<0.001

## 4. Discussion

Our analysis showed that anastomotic time is independently associated with the incidence of DGF with an odds ratio of 1.04 correlating with the fact that “every minute increment in anastomotic time increased the risk of DGF by 4%.”

Of the three ischemia times, the impact of AT is most sparingly studied. One of the initial papers to review the effect of vascular anastomosis was from Spain in 1991, which failed to demonstrate any detrimental effect on cadaveric renal graft survival (17). Later in that decade, a study from Japan by Hatsuse et al. in 1998 showed around 33% primary non-function in deceased donor transplant with more than 60 min of anastomotic time as compared to 11 and 4% in 30–60 min and less than 30 min of anastomotic time (18).

The last decade has seen a new interest in evaluating the significance of AT, but still, the overall available data were minimal. In 2013, Marzouk et al. in a retrospective analysis of 300 deceased donor transplants showed AT to be an independent factor affecting DGF with an increase in the odds ratio of 1.03 per minute, which is very similar to our results. They further showed that every 5 min of extra AT resulted in one extra day in the hospital and a higher day seven creatinine (2.0 μmol/L per minute) (19).

A more detailed analysis was published by Heylen et al. where they analyzed 669 renal transplantation from brain dead donors and showed a similar increase in the DGF odds ratio of 1.05 per minute. They also showed an increased risk of intestinal fibrosis and tubular atrophy on protocol biopsies at 3, 12, and 18 months post-transplant (20, 21). The same group conducted a Eurotransplant cohort study including 13,964 recipients and concluded a higher risk of graft loss from prolonged AT (10% per 10 min), with added negative impact on DCD kidneys and AT of >45 min (22).

Khan et al. reinstated similar findings and showed AT of >45 min as a risk factor for poor EGF but had a statistically significant difference only beyond 60 min (23). More recently, Ferede et al. confirmed similar findings with the increase in DGF with increasing AT but no difference in graft function at 3 months (24). AT and DGF have also been found to be of significance even in living donor transplants. In 2012, Hellengering et al. showed that prolonged WIT of more than 45 min in living donor transplants had a significant negative impact on early graft function and long-term graft survival (25).

In our study, DGF occurred in a significant percentage of DD transplants (44%) with a much higher incidence in DCD transplants (75%) as compared to DBD transplants (32%).

The adverse impact of DGF has been well established. A recently published study in 2021 showed DGF to be the greatest predictor of graft failure at 3 years, after analyzing UNOS/OPTN data of 42,736 living donor transplants (26).

Incerti et al. evaluated the overall lifetime health burden of DGF as a loss of 3.01 quality adjusted life-years for a typical 56-year-old transplant recipient with DGF as compared to a non-DGF recipient of the same age (12). DGF is also associated with an increased financial burden as reported by Kim et al. with approximately US $18,000 increase in mean cost and six additional days of hospital stay in patients with DGF (27).

Our sub-analysis of serum creatinine at 1 month and 1 year was found to be significantly different in the DGF and non-DGF groups. We did not compound the rejection data or biopsy findings in analyzing the graft function as it was not the primary aim of our study, but similar findings have been reported in other studies (4, 5, 8, 16, 28).

In our logistic regression analysis, CIT was not found to be significantly associated with DGF as opposed to the findings in various other studies (14, 29–32). This could be because the majority of our transplants have relatively shorter CIT, less than 16 h with a median of 840 min and narrow IQR of 660–980 min.

The factors influencing anastomotic time include the complexity of the surgery, number of arteries, number of warm anastomosis, body habitus of the recipient, previous transplants, and expertise of the surgeon. Based on our findings and existing evidence, all attempts should be made to reduce the AT along with utilizing techniques to maintain hypothermia during anastomosis.

Kidney warming speed is estimated to be around 0.48°C/min once out of ice, reaching >20°C for 30 min of AT (33). Protective effects of renal hypothermia are believed to be lost at the metabolic threshold around 15–18°C (34). Hameed et al. in 2018 have analyzed different techniques to ameliorate organ warming during anastomosis like simple surface cooling techniques with the immersion of the kidney in bags of ice slush and the application of ice jackets that incorporate their own internal cooling mechanism (34). They concluded that effective cooling of renal parenchyma could be achieved with these techniques, but higher quality studies are required to access its impact on graft function or patient outcomes.

The strength of our study is that we have analyzed the impact of AT as a continuous variable as compared to other studies which have described it as a threshold effect based on time interval groups. Our study signifies anastomotic time as an independent factor affecting immediate graft function and demonstrates the incremental effect of time taken for vascular anastomosis on the occurrence of DGF, with every minute adding to a 4% increase in the incidence of DGF in deceased donor transplants.

There are some limitations to this study. First, this is a retrospective review with a small cohort. In addition, there was variability in the surgical technique as the transplants were done by different surgeons across the study period.

## 5. Conclusion

Anastomotic time had a significant impact on the development of DGF and hence should be minimized. The impact is approximately 4% increment in the incidence of DGF with every minute increase in anastomotic time. Further study to address the factors leading to increased anastomotic time would help to identify the potentially modifiable factors to reduce DGF rates.

## Data availability statement

The raw data supporting the conclusions of this article will be made available by the authors, without undue reservation.

## Ethics statement

The studies involving human participants were reviewed and approved by the Hunter New England Human Research Ethics Committee. Written informed consent for participation was not required for this study in accordance with the national legislation and the institutional requirements.

## Author contributions

All authors listed have made a substantial, direct, and intellectual contribution to the work, and approved it for publication.
